# ﻿Morphology and phylogeny of *Nitzschianandorii* sp. nov. (Bacillariophyceae), a new small-celled lanceolate species from a post-mining reservoir

**DOI:** 10.3897/phytokeys.241.117406

**Published:** 2024-04-04

**Authors:** Rafał M. Olszyński, Piotr K. Zakrzewski, Frédéric Rimet, Julia Sulkowska, Łukasz Peszek, Joanna Żelazna-Wieczorek

**Affiliations:** 1 University of Lodz, Faculty of Biology and Environmental Protection, Department of Algology and Mycology, ul. Banacha 12/16, 90-237 Łódź, Poland; 2 UMR Carrtel, INRAE, Universit´e Savoie-Mont Blanc, 75bis av. de Corzent, FR74203 Thonon les Bains, France; 3 University of Lodz, Faculty of Biology and Environmental Protection, Department of Cytobiochemistry, ul. Pomorska 141/143, 90-236 Łódź, Poland; 4 Department of Agroecology and Forest Utilization, University of Rzeszów, ul. Ćwiklińskiej 1A, 35-601 Rzeszów, Poland

**Keywords:** Diatom, new species, *Nitzschia*, phylogeny, post-mining reservoir, taxonomy

## Abstract

Post-mining reservoirs are distinguished by characteristic environmental conditions where specific diatom communities can be observed. Reservoirs created as a part of the reclamation plan after human mining activities are marked by unique chemical and physical water parameters. In the course of research on the diatoms from Bogdałów reservoir, we examined the taxonomic and morphological diversity of Nitzschia taxa from the section Lanceolatae occurring in a post-mining lignite reservoir. Our study describes a new species of *Nitzschia* from a post-mining reservoir, *Nitzschianandorii* Olszyński, Zakrzewski & Żelazna-Wieczorek, **sp. nov.** Morphometry and morphology analyses of new species were performed with light and scanning electron microscopy. Chloroplast morphology analysis was conducted with differential interference contrast microscopy and confocal laser scanning microscopy. Molecular data from SSU 18S, *rbcL* and *psbC* sequences were obtained from cultures of this taxon. Differential diagnosis of *Nitzschianandorii* Olszyński, Zakrzewski & Żelazna-Wieczorek, **sp. nov.** with co-occurring taxa: *N.lacuum* and *N.alpinobacillum* was performed using morphological traits and nMDS analysis of the valves’ morphometry.

## ﻿Introduction

Post-mining reservoirs are unique and in many cases present extreme water environments where specific diatom communities can be observed (Olszyński et al. 2019). The mineral composition of the reservoir bottom and drainage water from mining operations may impact the chemical and physical parameters of water. In some circumstances, we can observe an influence on one particular factor e.g. chloride which provides conditions for the growth of stenotopic species (Żelazna-Wieczorek et al. 2015). Reservoirs created after human mining activities can also be a hot spot for the observation of teratological forms (Sienkiewicz et al. 2023), as well as a place of occurrence of a new diatom species for science (Żelazna-Wieczorek and Olszyński 2016; Olszyński and Żelazna-Wieczorek 2018).

*Nitzschia* Hassall, 1845 (Bacillariophyceae) is one of the most numerous and various non-monophyletic genera where several phylogenetic clades and morphological sections can be distinguished (Rimet et al. 2011; Mann et al. 2021; Guiry 2023). According to the AlgaeBase, there are more than 880 registered *Nitzschia* species (Guiry 2023). Despite a large number of identified *Nitzschia* taxa, there is still a relatively small number of records of *Nitzschia* species sequences. According to the GenBank database (http://www.ncbi.nlm.nih.gov), sequences have only been submitted for 93 identified species (accessed 15 December 2023, identified to the species level). The Lanceolatae is one of the most diverse sections of *Nitzschia*, according to the Grunowian classification which is characterized by a strongly eccentric keel position, absence of longitudinal fold, linear and lanceolate valves and distinctive fibulae (Cleve and Grunow 1880). However, significant morphological diversity also exists in this section: in fibulae morphology, the presence or absence of the wider relative spacing between the central fibulae compared to the rest of the fibulae, diversified raphe canal structure, and the presence or absence of distinct proximal raphe fissures. Recent studies based on phylogeny analysis using *rbcL* marker revealed in the Lanceolatae, a clade of *Nitzschia* including *N.fonticola* (Grunow) Grunow, 1881, *N.perminuta* Grunow, 1881, *N.costei*Tudesque et al., 2008, *N.soratensis* E.A. Morales & M.L. Vis, 2007, and *N.acidoclinata* Lange-Bertalot, 1976 (Rimet et al. 2011; Mann et al. 2021). These species possess a specific shared morphology: two or more longitudinal rows of areolae on the raphe canal, a single row of poroids on the girdle band, no poroids on the second and third and elongated poroids on the fourth (Mann et al. 2021).

This paper aims to study the taxonomical and morphological diversity of Nitzschia taxa from the Lanceolatae section found in a studied lignite post-mining reservoir. Based on multi-threaded analyses which include the molecular analysis of three DNA markers--those encoding the small-subunit of ribosomes (SSU 18S rDNA), and chloroplast-encoded genes *rbcL* and *psbC*-- plus morphometry and morphology using light (LM) and scanning electron microscopy (SEM), including details of chloroplast morphology using differential interference contrast microscopy (DIC) and confocal laser scanning microscopy (CLSM), we have described *Nitzschianandorii* sp. nov.

## ﻿Materials and methods

### ﻿Study sites description

**Figure 1.**
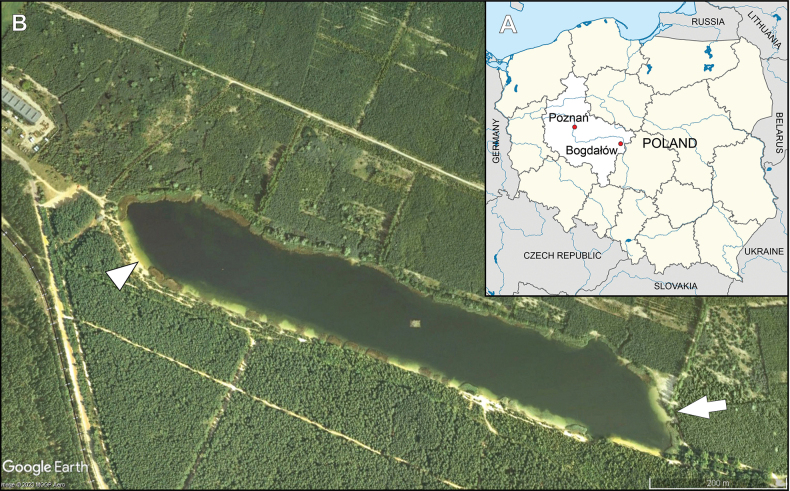
Location of studied Bogdałów reservoir **A** location of Greater Poland Voivodeship in Poland and Bogdałów village in Greater Poland Voivodeship **B** location of the sampling site, white arrowhead sampling site, white arrow inflow of stream (http://earth.google.com).

Samples were collected from the Bogdałów reservoir which is located in Bogdałów village in Greater Poland Voivodeship near the city of Turek (Fig. 1). This area is a part of the mining operation belonging to the PAK KWB Konin S.A. mining company. There are several lignite and brown coal opencast mines present, and several reservoirs created after flooded mining by water from local streams and mine drainage water. The reservoir was created after a flooded opencast mine of lignite in the middle of the 1990s as a part of the reclamation plan. The water surface is 10.8 ha, designed to store water for fire-fighting purposes, and is surrounded by forest (Orlikowski and Szwed 2009, 2011; Stachowski et al. 2018). In 2019, the first studies on diatom communities in this area were conducted to verify or establish ecological indicators values (Olszyński et al. 2019).

### ﻿Sampling and culture

The study site (52°2'53.938"N, 18°35'49.646"E) is located on the opposite side of the inflow stream supplying the reservoir to avoid the direct impact of the variability of disturbance caused by lotic water (Fig. 1). Four samples were taken from the sandy bottom, 10 m of the reservoir shore at a depth of around one meter and from the submerged vascular plants in October 2022 by the glass pipette (sand) and toothbrush (plants) and transferred to 100 mL containers. Epipsammic samples were collected only from the surface layer to take live diatom cells. Temperature, pH, and conductivity were measured *in situ* by using CC-411 and CP-401 devices (ELMETRON, Poland). Samples were taken to the laboratory of the Department of Algology and Mycology, University of Lodz for further processing and analysis. Single diatom cells were isolated with a glass micropipette using a Nikon Eclipse TS100 E Plan APO 10x/0.25 WD 7.0 inverted light microscope (Nikon, Japan). Cells were transferred to sterile plastic Petri dishes (6 cm diameter) and enriched with sterile Bacillariophycean Medium (= Diat.) ver. 10.2008 (Sammlung von Algenkulturen Göttingen Medium Recipe Culture Collection of Algae) and cultivated 12/12 photoperiod to obtain monocultures. Monoclonal strains were transferred to cell culture bottles, 25 cm^2^ with 20 mL Diat. medium and cultivated 12/12 photoperiod at 22 °C. Two strains – D.LDZ.8 and D.LDZ.12 – were selected for further analyses. To analyze the morphological features of diatoms of the genus *Nitzschia* and also to describe ecology and the water ecological status of Bogdałów reservoir, data from the eight samples collected during previous research from March 2015 to December 2016 (Olszyński et al. 2019) were taken to calculate IPS (CEMAGREF 1982) and IBD 2014 (Coste et al. 2009) indices using OMNIDIA 6.0.6 software (Bordeaux, France).

### ﻿Microscopy analysis

To obtain cleaned diatom frustules, the environmental and cultured samples were treated according to the procedure outlined by Żelazna-Wieczorek (2011). The purified material was applied to coverslips and, after drying, mounted to glass microscope slides in Naphrax^®^ resin (Brunel Microscopes Ltd., UK). The taxonomic, and morphometric analyses were performed with a Nikon Eclipse E400 microscope with 1000× magnification (plan oil-immersion objective 100×/1.40 Plan APO DIC H) and a NIKON DS-L1 digital camera (Nikon, Japan) was used to obtain photomicrographs of cleaned frustules from recent and previous samples. For SEM, a few drops of cleaned material were placed on Whatman Nuclepore polycarbonate membrane filters (5 µm pore diameter) (Fisher Scientific, Germany). Once dried, the membranes were mounted on aluminium stubs and coated with 20 nm of gold using a turbo-pumped Quorum Q 150T ES coater (Judges Scientific plc, UK). SEM observations were performed using a Hitachi SU8010 (Hitachi, Ltd, Japan) microscope. To obtain photomicrographs of chloroplasts from living cultured cells, confocal laser scanning microscopy (CLSM) with 3D imaging was performed using Leica SP8 3D visualization (Leica Camera AG, Germany).

### ﻿Morphometric analysis

For the morphometric analysis, 83 valves of three different *Nitzschia* taxa were described and measured, using only light microscopy, by six different traits: (1) width, (2) length, (3) L/W ratio, number of (4) striae and (5) fibulae in 10 µm, if the number of striae was indistinguishable we assumed more than 30 striae in 10 µm, (6) presence =1 or absence = 0 of wider interspace between centre fibulae, and (7) shape of the apices (subcapitate = 1, capitate = 2) (see Suppl. material 1). Morphometric analysis was performed based on two environmental samples: benthic collected in October 2015 (Olszyński et al. 2019) and the second obtained from vascular plants in October 2022. All three species occurred together in each sample. Based on the Euclidian distance, non-metric MDS analysis (nMDS) performed using PRIMER 7 (PRIMER-E Ltd, UK) software we evaluated morphometrical differences between study specimens. For the analysis of the ultrastructure of the valves and count of more than 30 striae in 10 μm in studied species, SEM was used.

### ﻿DNA isolation, amplification, and sequencing

Total DNA isolation from diatom monoclonal cultures was performed using the Chelex^®^ 100 (Bio-Rad, CA, USA) method described by Dąbek et al. (2017). The diatom biomass was obtained from 1.5 mL of cell suspension after centrifugation for 5 min at 5,000 rpm. This pellet was incubated with 200 µl of 10% Chelex^®^ 100 working solution at 95 °C for 20 min with continuous shaking. Next, samples were centrifuged for 5 min at 5,000 rpm to remove cell debris and Chelex^®^ 100 resin. The DNA-containing supernatant was transferred to a new sterile 1.5 mL Eppendorf^®^ tube and stored at -20 °C for further analysis. Three molecular DNA markers, i.e. nuclear (SSU 18S) and chloroplast (*rbcL* and *psbC*) were amplified in the PCR using primers listed in Table 1 (Kawai et al. 2015; Theriot et al. 2015). PCR was conducted in a T100 Thermal Cycler (Bio-Rad, CA, USA) using HOT FIREPol^®^ DNA Polymerase (Solis BioDyne OÜ, Estonia) according to the manufacturer’s protocol. Briefly, PCR amplifications were conducted in a total volume of 25 µl, consisting of 0.15 µl HOT FIREPol^®^ DNA polymerase (5U/µl), 2.5 µl HOT FIREPol^®^ 10x Buffer B1, 2 µl MgCl_2_ (25 mM), 0.5 µl primer F, 0.5 µl primer R, 0.25 µl dNTPMix (20 mM each), and H_2_O nuc.-free up to 22 µl. The 3 µl of Chelex^®^ 100 treated supernatant served as a template for amplification. Thermal conditions were as follows: initial denaturation at 95 °C for 15 min, followed by 35 cycles of sequential incubations at 95 °C for 30 s; at 50 °C (SSU) or 56 °C (*rbcL*), or 57 °C (*psbC*) for 1 min.; at 72 °C for 60s (SSU) or 90 s (*rbcL*, *psbC*); at 72 °C for 10 min. All markers were amplified by PCR in a few replicates to obtain high-quality products, which in turn were visualized on 1% agarose gel (Sigma-Aldrich, Germany) and stained with ethidium bromide (Sigma-Aldrich, Germany). PCR products were cleaned up and sequenced with Sanger sequencing, using primers used for amplification by SEQme s.r.o. company (Czech Republic). The obtained sequences were assembled in Geneious Prime^®^ 2023.2.1 (Biomatters Inc., MA, USA) and submitted to the NCBI GenBank database (http://www.ncbi.nlm.nih.gov).

**Table 1.** Characteristics of primers used for phylogenetic studies.

Marker/ Forward/reverse primer; name (sequence)	T_m_	Author
**SSU 18S**		
SSU_F (5’-TGTAAAACGGCCAGTATTCCAGCTCCAATAGCG-3’)	50 °C	this study
SSU_R (5’-CAGGAAACAGCTATGACGACTACGATGGTATCTAATC-3’)		
***rbcL***		
rbcL40 (5’-GGACTCGAATYAAAAGTGACCG-3’)	56 °C	Theriot et al. 2015
rbcL1444 (5’-GCGAAATCAGCTGTATCTGTWG-3’)		
***psbC***		
psbC-P1.2 (5’-CCACGTGGAAACGCYCTTTA-3’)	57 °C	Kawai et al. 2015
psbC-P3 (5’-CTTGCCAAGGTTGRATATCATT-3’)		

### ﻿Phylogenetic analyses

The phylogenetic analyses on studied samples and other species of the *Nitzschia* genus were performed separately for each marker, i.e. SSU, *rbcL*, and *psbC*, as well as based on a concatenated SSU-*rbcL*-*psbC* matrix (2236 bp). *Eunotiabilunaris* (Ehrenberg) Schaarschmidt, 1880 served as an outgroup. For taxa in the phylogenetic analysis with incomplete gene sampling, such regions were treated as ‘missing data’. Voucher information for the specimens included in this study, with corresponding GenBank accession numbers, are presented in Suppl. material 2. The performed analyses included 60, 116, and 37 sequences, respectively for SSU, *rbcL* and *psbC* markers. Sequences were aligned using the MAFFT v. 7 web server (Katoh et al. 2019) (http://mafft.cbrc.jp/alignment/server/) where the auto strategy was applied, the scoring matrix of 200PAM with Gap opening penalty of 1.53, UniREf50 for Maft-homologs and Plot and alignment with a threshold of 39 score were set (see Suppl. materials 3–6). The obtained alignments were checked for poorly and ambiguously aligned regions and small corrections were made by eye. The evolutionary models were calculated using Mega 11 Version 11.0.13 and were chosen according to the Akaike Information Criterion. The dataset was partitioned by gene and codon position (*rbcL* and *psbC*), whereas the SSU marker was left unpartitioned. The partitioning was performed using PartitionFinder2 software (Lanfear et al. 2016; see: Suppl. material 7). Phylogenetic calculations were performed using maximum likelihood analysis (ML) in the IQ-TREE web server (Trifinopoulos et al. 2016) (http://iqtree.cibiv.univie.ac.at/) with the ultrafast bootstrap (UFBoot) pseudolikelihood algorithm (Hoang et al. 2018) and 10000 replicates; and Bayesian inference (BI) in MrBayes 3.2.7 (Ronquist et al. 2012) which provides Markov chain Monte Carlo (MCMC) method to estimate the posterior distribution of model parameters. Trees were sampled every 1000 generations until the average standard deviation of split frequencies reached values below 0.01 for the last 1000 generations and posterior probabilities were estimated from the 50% majority-rule consensus tree after elimination of the first 25% of samples as burn-in.

## ﻿Results

### ﻿Environment background

Measurement of parameters collected in October 2022 indicates a slightly alkaline water reaction (pH = 7.98) with conductivity of 658 µS·cm^-1^ and temperature of 11.2 °C. The ecological status of water quality in Bogdałów reservoir based on IPS and IBD 2014 indices (calculated from previous research) varied and depended on the season (Olszyński et al. 2019). During the summer and autumn months, moderate to poor conditions existed, while in winter and spring moderate to very good ecological statuses of the water were noted. Based on Van Dam et al. (1994), this environment can be classified as freshwater and alkalic with high oxygen saturation, from meso-eutrophic to oligotrophic and β-mesosaprobic to oligosaprobic (Olszyński et al. 2019).

#### ﻿ Nitzschia nandorii

Taxon classificationPlantaeCapsalideaCapsalidae

Olszyński, Zakrzewski & Żelazna-Wieczorek sp. nov.

76218E52-AED5-5ECF-9972-4C572630D3AE

**Figure 2.**
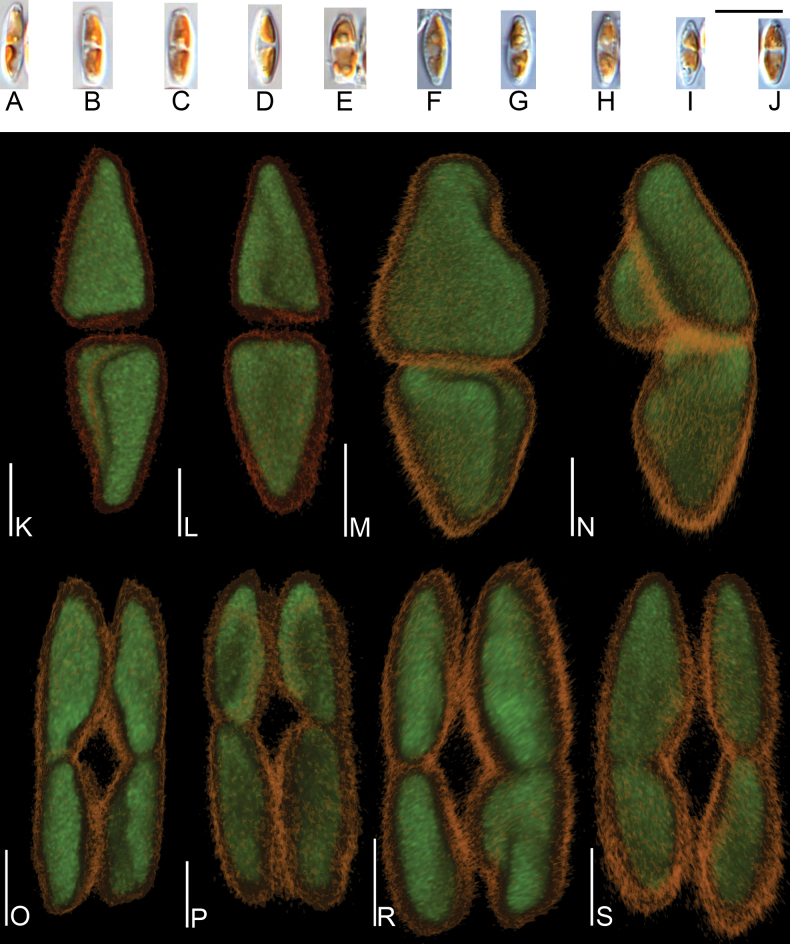
Photomicrographs of chloroplast *Nitzschianandorii* sp. nov. **A–J** light microscope with DIC photomicrographs **K–S** Confocal Laser Scanning Microscopy photomicrographs, each pair of photomicrographs presents the same chloroplasts from different angles **K–N** presented single-cell, conical-shaped chloroplasts with visible longitudinal groves **O–S** presented chloroplasts during cell division. Scale bars: 10 µm (**A–J**); 2 µm (**K–S**).

**Figure 3.**
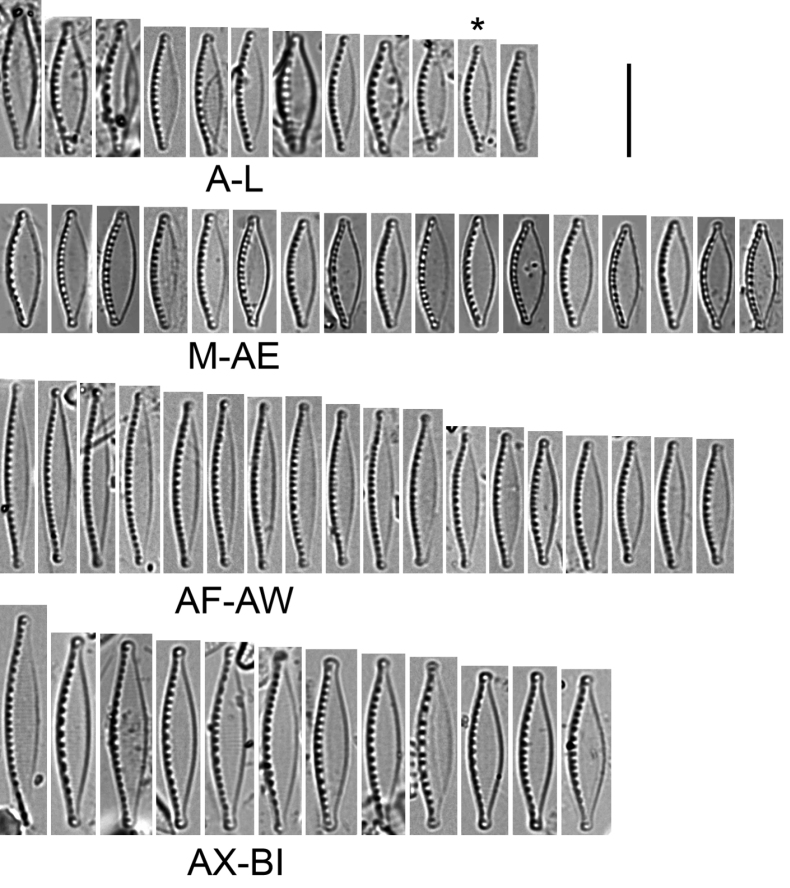
Light microscope photomicrographs of *Nitzschia* spp. **A–L***N.nandorii* sp. nov. from natural population **K** type species (marked by an asterisk (*)) **M–AE***N.nandorii* sp. nov. from culture **AF–AW***N.lacuum***AX–BI***N.alpinobacillum*. Scale bar: 10 µm.

Figs 2 , 3A–AE , 4 –6

##### Holotype.

Slide number: D.BOF2.191022, Algae Collection Department of Algology and Mycology, University of Lodz. The holotype is illustrated in Fig. 3K (designated here).

##### Isotype.

Slide number: SZCZ 29106, Szczecin Diatomological Collection, University of Szczecin, Poland.

##### Type locality.

Poland. Greater Poland Voivodeship, Bogdałów. Post-mining reservoir Bogdałów. 52°2'53.938"N, 18°35'49.646"E.

##### Morphology description.

**Figure 4.**
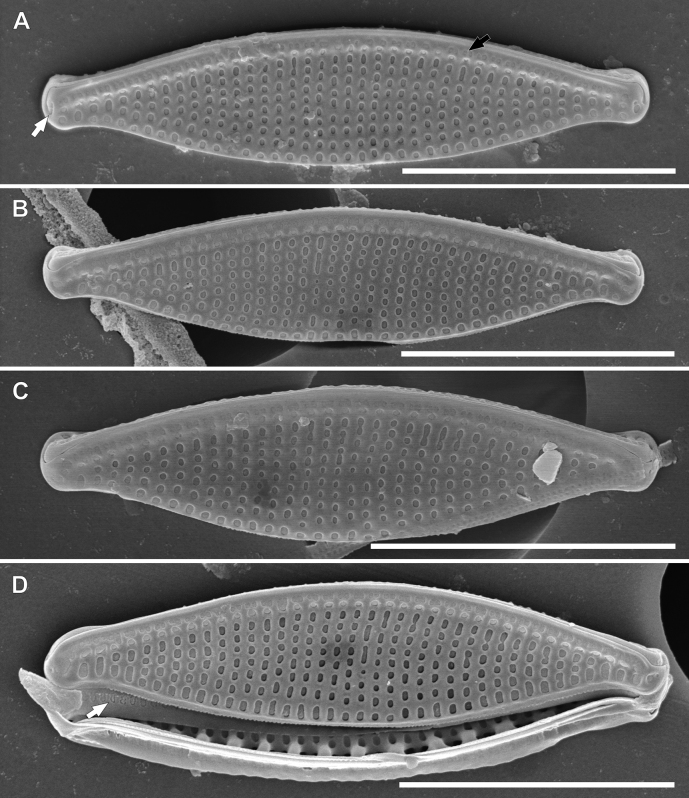
SEM photomicrographs of *Nitzschianandorii* sp. nov. external valve view **A** valve view with two rows of areolae on the raphe canal (black arrow), single and double areolae, distal raphe fissures are strongly hooked and deflect (white arrow) **B, C** valve view with distal raphe fissures deflected to the proximal mantle **D** decomposed frustule with visible valvocopula with open pores (white arrow). Scale bars: 5 µm.

**Figure 5.**
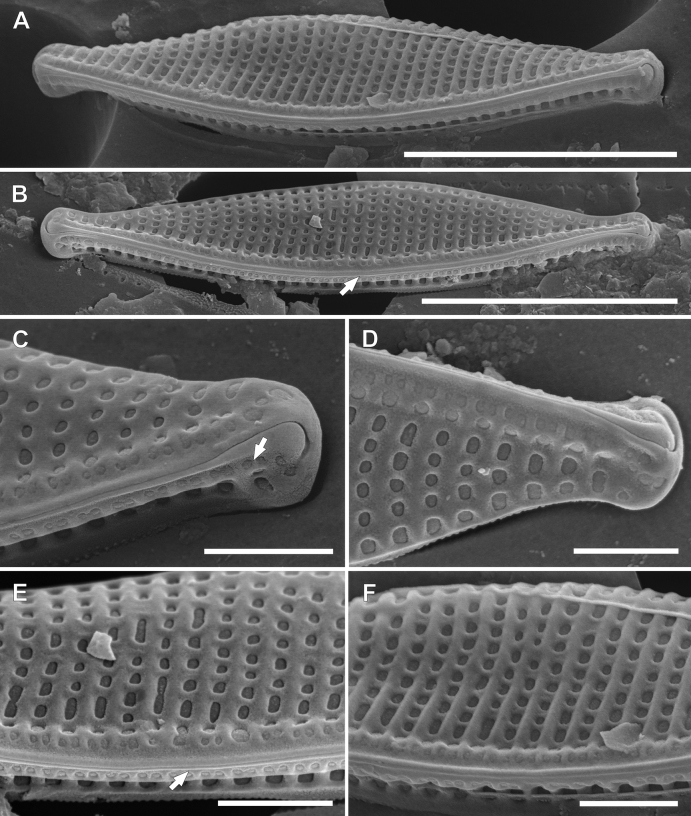
SEM photomicrographs of *Nitzschianandorii* sp. nov. external view **A, B** angle view with visible keel **B** white arrow indicates uninterrupted raphe **C, D** apices view with distal raphe fissures deflected to the opposite direction **C** white arrow indicates rows of areolae on proximal mantle **E, F** close-up central part of the frustule **E** white arrow indicates missing of central raphe fissures. Scale bars: 5 µm (**A, B**); 1 µm (**C–F**).

**Figure 6.**
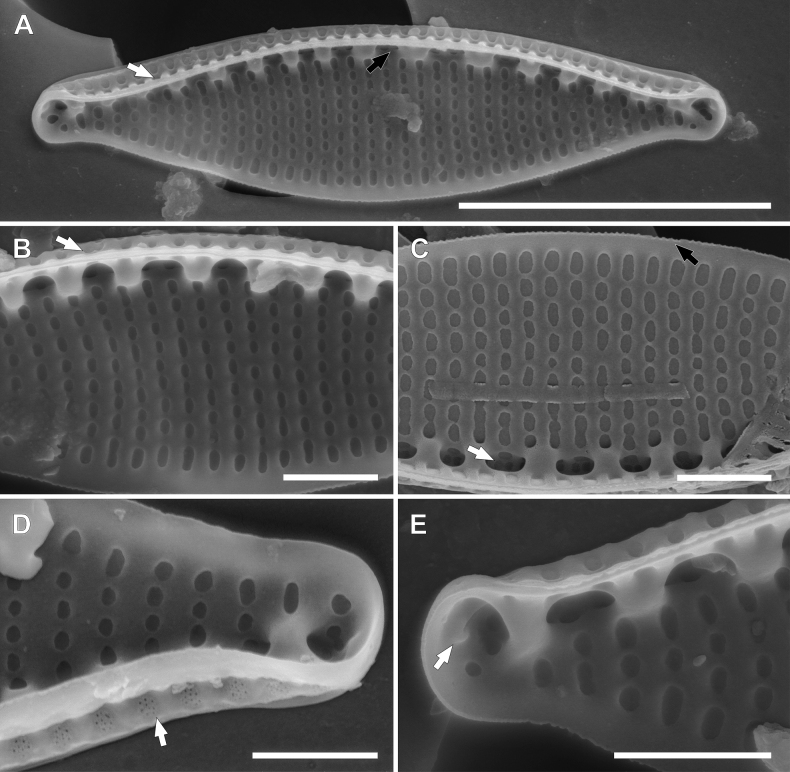
SEM photomicrographs of *Nitzschianandorii* sp. nov. internal view **A** valve view with areolae extended on the proximal mantle (white arrow), there is no wider extension between central fibulae (black arrow) **B, C** central view **B** areolae on the proximal mantle are arranged the same as on the raphe canal: single and double areolae (white arrow) **C** doubled areolae are visible from inside the raphe canal (white arrow), narrow mantle with scalloped edge (black arrow) **D, E** apices view **D** areolae on the mantle covered by hymenes (white arrow) **E** distal fissure creates helictoglossa (white arrow). Scale bars: 5 µm (**A**); 1 µm (**B–E**).

*Nitzschianandorii* sp. nov. possesses two conical-shaped chloroplasts arranged apically with a central, longitudinal groove (Fig. 2, see: Suppl. material 8). Frustules are small, distended, and broadly lanceolate with short protracted and subcapitate apices. Valves are 9.0–12.0 µm length and 2.5–3.3 µm width. The ratio of length and width is 3.44–4.80 (Fig. 3A–AE). Striae 40–46 in 10 µm (based on SEM analysis) and indiscernible in LM. Fibulae are visible 11–16 in 10 µm. Externally, the valve face is flat and without costae (Figs 4, 5). Striae are uniseriate, and transapically becoming more arched to the apices (Figs 4, 5). Two longitudinal rows of areolae are present along the edge of the valve on the raphe canal with a doublet of pores closest to the raphe and second with a single pore near the junction of the raphe canal and valve face (Fig. 4). Distal raphe endings are strongly hooked and deflected in different directions depending on the valve. Polar raphe fissures deflected to the proximal mantle overlap on it (Figs 4, 5A–D). The central raphe fissures are missing (Fig. 5A, B, E, F, see: Suppl. material 9). The proximal mantle possesses three rows of areolae (Figs 5C, 6B, see: Suppl. material 9). The first two are arranged to mirror pores on the raphe canal and open to the raphe canal (Fig. 5C). The third with areolae covered by hymenes (Fig. 6D) and open to frustule interior (Fig. 5C, see: Suppl. material 9). The distal mantle is narrow with a scalloped edge (Figs 4D, 6C). Valvocopula is smooth and possesses opened pores (Fig. 4D). Internally, the fibulae are roughly equidistant so that the central fibulae are not further apart than any of the others (Fig. 6A). Distal raphe fissures terminate with small helictoglossa internally (Fig. 6E).

##### Gene sequences.

sequences were deposited in the GenBank: D.LDZ8 (SSU rDNA: PP082029, *rbcL*: PP073739, and *psbC*: PP073738), D.LDZ12 (SSU rDNA: PP082030, *rbcL*: PP073741, and *psbC*: PP073740)

##### PhycoBank registration.

http://phycobank.org/104257.

##### Molecular phylogeny.

**Figure 7.**
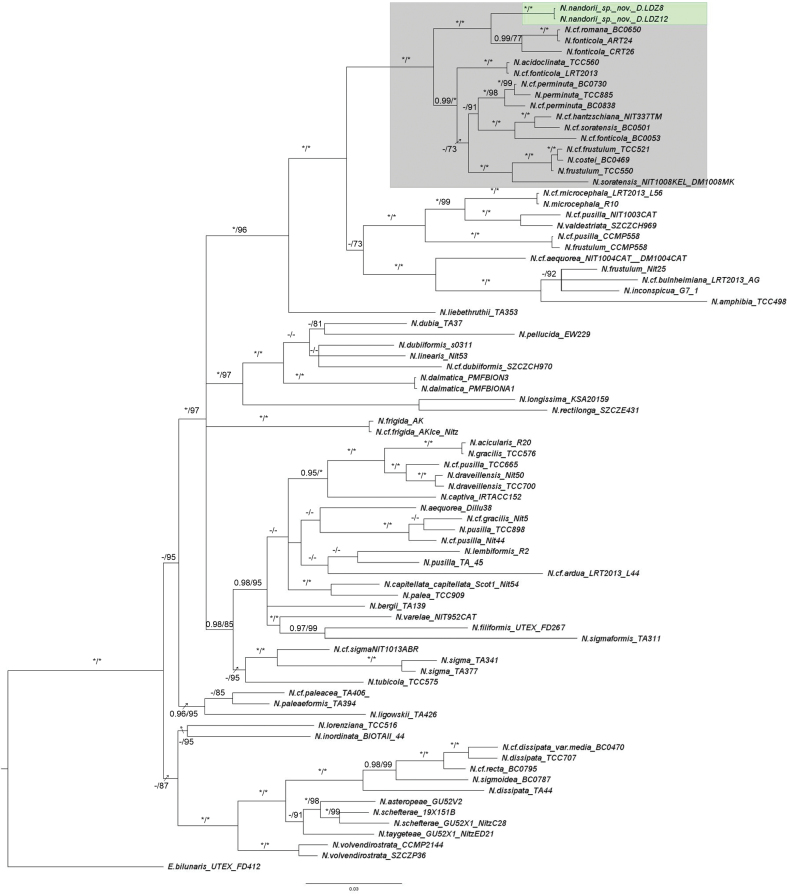
The phylogenetic tree of the *Nitzschia* genus with *Eunotiabilunaris* as the outgroup based on the concatenated nuclear (SSU) and chloroplast (*rbcL* and *psbC*) DNA markers (total 2236 bp). The tree presents the position of newly identified *Nitzschianandorii* sp. nov. The numbers above branches represent posterior probabilities from BI analysis followed by bootstrap values from ML analysis. Asterisk (*) represents BI value = 1, and ML value = 100. En dash (-) represents BI value below 0.95, and ML value below 70. Grey box: Lanceolatae section with double rows of areolae on the raphe canal. The topology of the tree is based on BI analysis.

**Figure 8.**
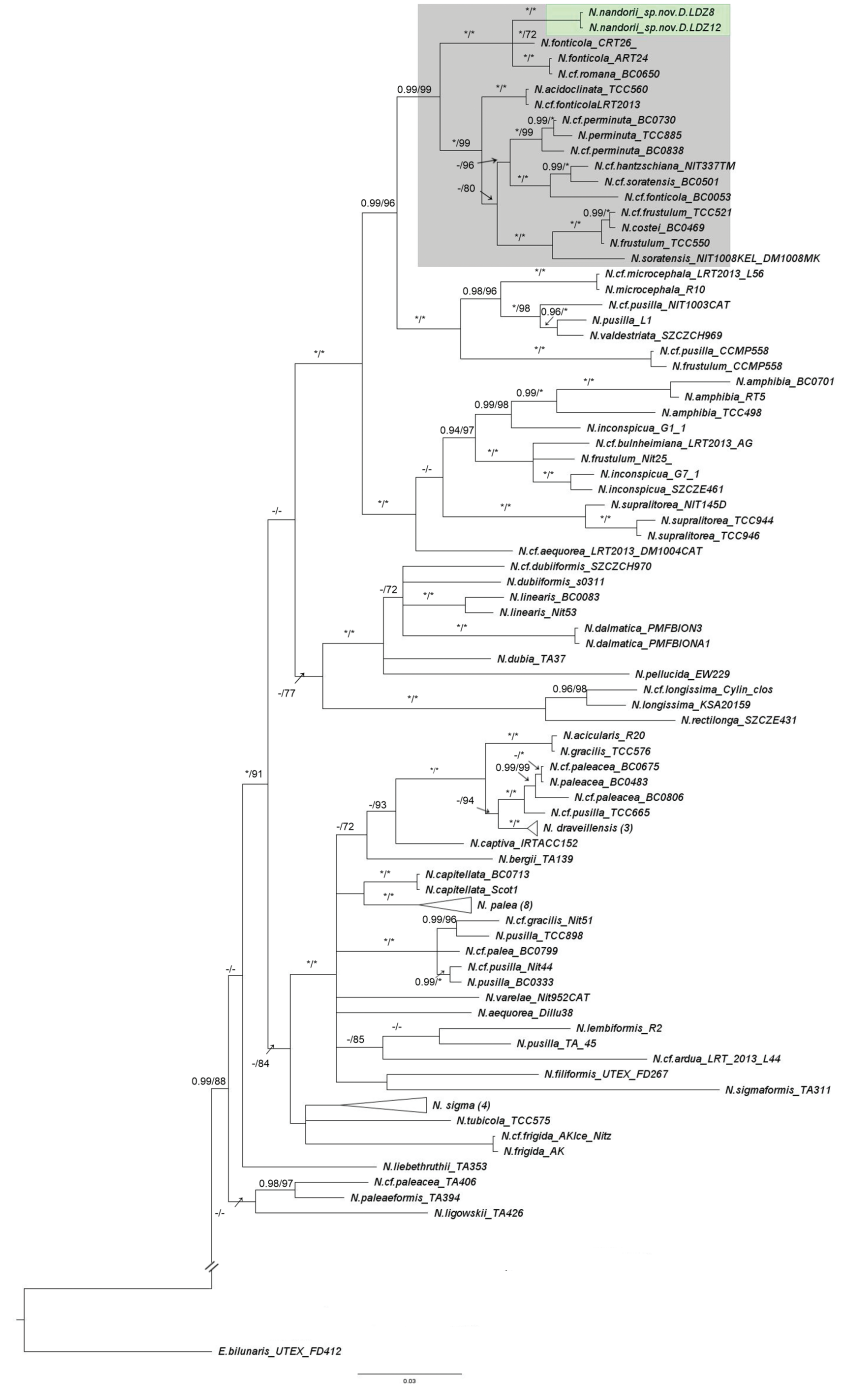
The phylogenetic tree of the *Nitzschia* genus with *Eunotiabilunaris* as the outgroup based on chloroplast (*rbcL*) DNA marker (1147 bp). The tree presents the position of newly identified *Nitzschianandorii* sp. nov. The numbers above branches represent posterior probabilities from BI analysis followed by bootstrap values from ML analysis. Asterisk (*) represents BI value = 1, and ML value = 100. En dash (-) represents BI value below 0.95, and ML value below 70. Grey box: Lanceolatae section with double rows of areolae on the raphe canal. The topology of the tree is based on BI analysis.

The phylogenetic reconstructions based on the ML and BI strategy for the concatenated SSU-*rbcL*-*psbC* matrix, as well as separate SSU, *rbcL*, and *psbC* analysis, were performed. The three-genes tree placed strains D.LDZ8 and D.LDZ12 (Fig. 7, green box) within the clade including the Lanceolatae section, which is characterized by double rows of areolae on the raphe canal, with very high node support (BI = 1; ML = 100) (Fig. 7, grey box). Moreover, the tree topology distinguished the strains described here, i.e., *N.nandorii* sp. nov., from *N.fonticola* (N.cf.romana syn. *N.fonticola*) both in BI and ML phylogenetic reconstructions with the strong division support (BI = 1; ML = 100). Phylogenetic trees reconstructed using separate DNA markers, i.e., *rbcL* (Fig. 8), *psbC* (see Suppl. materials 10, 11), and SSU (see Suppl. materials 12, 13), and the concatenated analysis distinct *N.nandorii* sp. nov., as a separate clade in Lanceolate section. The *rbcL* tree topology (Fig. 8) was included in the main manuscript here, due to its higher taxon sampling.

##### Etymology.

The species name comes from the main character of the TV series “*What We Do in the Shadows*” Nandor the Relentless, and the name of the authors’ cat (RMO, PKZ).

##### Differential diagnosis.

*Nitzschianandorii* sp. nov. is a species with a small cell size that can be overlooked or misidentified with other taxa, especially when they occur in one sample. The valve shape of the small specimens of *N.fonticola* can be misidentified with *N.nandorii* sp. nov., however visible striation and the wider relative spacing between the central fibulae compared to the rest of the fibulae in *N.fonticola* distinguish these two taxa (Lange-Bertalot 1976; Krammer and Lange-Bertalot 1997; Lange-Bertalot et al. 2017). The valve outline of *N.dealpina* Lange-Bertalot & G. Hofmann 1993 is similar to *N.nandorii* sp. nov., but these taxa can be distinguished due to the lower stria and fibula density per 10 µm in *N.dealpina*. Furthermore, *N.dealpina* occurs in an environment with a high concentration of calcium bicarbonate while *N.nandorii* has so far only been identified in an environment with a moderate concentration of these ions (Lange-Bertalot 1993; Krammer and Lange-Bertalot 1997; Rumrich et al. 2000; Lange-Bertalot et al. 2017; Olszyński et al. 2019). The smaller forms of *N.alpinobacillum* Lange-Bertalot, 1993, which also possess two rows of pores on the raphe canal (Lange-Bertalot 1993; Rumrich et al. 2000; Lange-Bertalot et al. 2017), have similar outlines to the *N.nandorii* sp. nov., but have denser striation and fibulae. *N.nandorii* sp. nov. can be easily misidentified with a small *N.lacuum*Lange-Bertalot 1980. Both taxa have similar valve size and stria and fibula density. Neither has a central nodule or a relatively wide gap between central fibulae. However, *N.lacuum* has different proportions between length and width and, therefore, is more elongated and has more protracted apices than *N.nandorii* sp. nov. (Lange-Bertalot 1980, 1993; Lange-Bertalot et al. 2017). *N.bryophila* (Hustedt) Hustedt 1943 has a similar valve outline to *N.nandorii* sp. nov. However, the frustules of *N.bryophila* are bigger, apices are more elongated and have clearly visible striations. Fibulae are less dense in *N.bryophila* than in *N.nandorii* sp. nov. (Lange-Bertalot and Metzeltin 1996; Krammer and Lange-Bertalot 1997). *N.bacillum* Husted, 1922 has a similar valve outline to *N.nandorii* sp. nov. In some cases a small form of *N.bacillum* can be misidentified with newly described species. However, *N.bacillum* has visible striations and particular areolae can be observed. A character which is visible in SEM and distinguishes this species from *N.nandorii* sp. nov. is a single row of areolae on the raphe canal (Lange-Bertalot 1993). Another similar species to *N.nandorii* sp. nov. is *N.rosenstockii* Lange-Bertalot, 1980. This taxa has specific apices which are short, beak-like and point rounded. In some cases, the valve outline can be similar to *N.nandorii* sp. nov. However, *N.rosenstockii* can be easily distinguished in LM from *N.nandorii* sp. nov. by the valve outline, characteristic valve ends, a higher number of fibulae in 10 µm, and the presence of striae discontinuity in the central part of valves (Lange-Bertalot 1980). For more details see Table 2.

**Table 2.** Comparison of morphological features of *Nitzschianandorii* sp. nov. with similar species. * literature data, n/d – no data.

	Valve outline	Valve length [µm]	Valve width [µm]	Number of striae in 10 µm	Number of fibulae in 10 µm	Length/width ratio	Additional features	Reference
*N.nandorii* sp. nov.	Distended, widely lanceolate with short protracted subcapitate ends	9.0–12.0 (n = 19)	2.6–3.3 (n = 19)	40–46 (n = 17)	14–16 (n = 19)	3.44–4.80 (n = 19)	All fibulae are relatively equidistant, and central fissures missing. Two rows of striae on raphe canal	This paper
*N.lacuum*	Lanceolate with capitate to acutely rounded ends	13.0–22.0 (n = 48) 10.0–20.0*	2.0–3.5 (n = 48) 2.0–3.0*	36–38 (n = 11) 35–40*	13–18 (n = 48)	4.38–10.00 (n = 48)	All fibulae are relatively equidistant, and central fissures missing.	This paper; Krammer and Lange-Bertalot 1997; Lange-Bertalot et al. 2017
*N.alpinobacillum*	Lanceolate with elongated and capitate ends	15.0–22.0 (n = 16) 14.0–24.0*	3.0–4.0 (n = 16) 3.0–4.0*	23–24 (n = 16) 25–27*	10–14 (n = 16) 9–11*	4.21–6.41 (n = 16)	All fibulae are relatively equidistant. Two rows of striae on raphe canal	This paper; Lange-Bertalot 1993; Lange-Bertalot et al. 2017
*N.dealpina*	Relatively short, appearing more widely lanceolate with short protracted and acutely rounded	8.0–13.0*	3.2–4.2*	26–28*	12–14*	n/d	No wider extension between central fibulae. One row of striae on raphe canal	Lange-Bertalot and Metzeltin 1996
*N.fonticola*	Widely or narrowly lanceolate with drawn-out, subcapitate ends	7.0–46.0*	2.5–5.5*	24–33*	9–14*	n/d	Relatively wide gap between central fibulae	Krammer and Lange-Bertalot 1997; Lange-Bertalot 1976
*N.bryophila*	Lanceolate to linear-lanceolate with short or slightly capitate ends	15.0–26.5*	4.0–5.0*	30–32*	9–10*	n/d	No wider extension between central fibulae.	Lange-Bertalot and Metzeltin 1996; Krammer and Lange-Bertalot 1997
*N.bacillum*	Lanceolate with pointed or slightly capitate ends	12.0–20.0*	2.0–3.5.0*	27–32*	12–16*	n/d	No wider extension between central fibulae. One row of striae on raphe canal	Lange-Bertalot 1993; Krammer and Lange-Bertalot 1997
*N.rosenstockii*	Lanceolate with short, beak-like, pointed rounded ends	8.0–16.0*	3.0–4.0*	44–46	17–20	n/d	No wider extension between central fibulae. Hyaline area in centre of the valve	Lange-Bertalot 1980; Krammer and Lange-Bertalot 1997

#### ﻿ Nitzschia lacuum

Taxon classificationPlantaeCapsalideaCapsalidae

Lange-Bertalot, 1980

66EB7EA8-755A-5A10-8060-273726B6782C

Figs 3AF–AW , 9

##### Notes.

**Figure 9.**
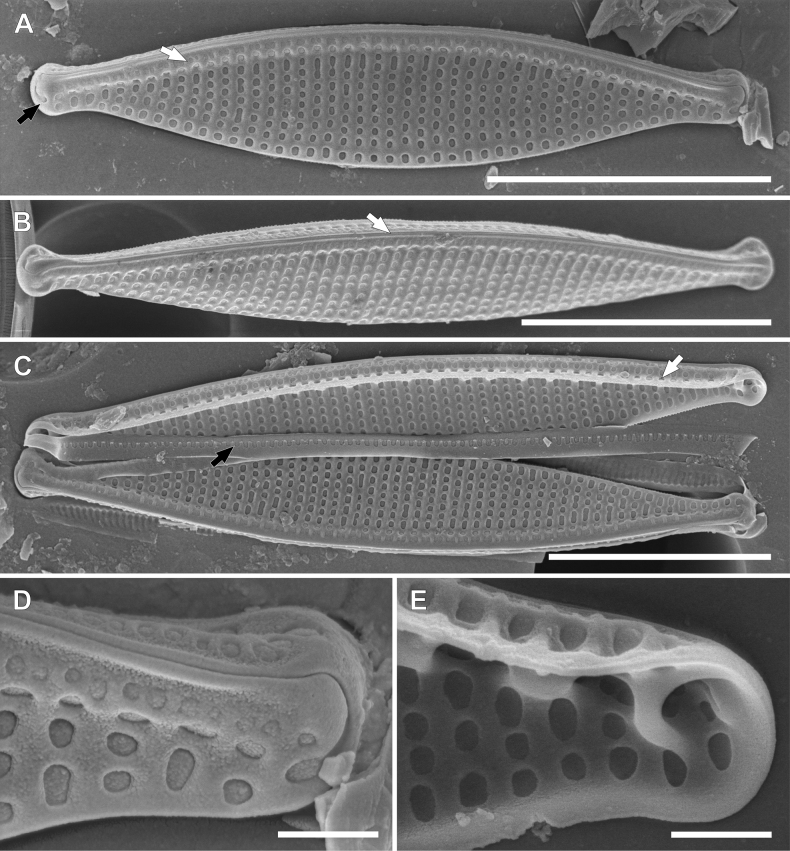
SEM photomicrographs of *Nitzschialacuum***A–C** valve view **A** visible two rows of areolae on the raphe canal (white arrow), distal raphe fissures are strongly hooked and deflect (black arrow) **B** frustule with a visible proximal mantle, the arrangement of areolae in the proximal mantle is the same as in the raphe canal, central view with raphe without central fissures, the small ridge on the raphe canal (white arrow) **C** decompose frustule, proximal mantle with areole and at least one open into the raphe canal (white arrow), valvocopula with scalloped edges (black arrow) **D** apex with strongly hooked distal raphe fissure **E** internal view of the apex with visible distal raphe fissure terminate with helictoglossa. Scale bars: 5 µm (**A–C**); 0.5 µm (**D, E**).

The outline of the frustules is lanceolate with capitate to acutely rounded apices (Fig. 3AF–AW). Valves are 13–22 µm length and 2.0–3.5 µm in width. The ratio of length and width is 4.38–10.00. Striae 36–37 in 10 µm (undistinguished in LM). Fibulae are visible 11–18 in 10 µm. Striae are uniseriate, arranged transapically. Along the edge of the valve, where the raised raphe canal is located, two longitudinal rows of areolae are present (Fig. 9A, D) – a doublet nearer to the raphe and a single areola at the junction with the valve face opened to the interior of the raphe canal. Distal raphe endings are strongly hooked and deflect on each valve in different directions (Fig. 9A, B, D). The central nodule is absent (Fig. 9B). The proximal mantle possesses three rows of areolae (Fig. 9C). Valvocopula possess scalloped edges (opened pores) (Fig. 9C). The distal mantle is narrow and has scallops on the edge (Fig. 9B, C). Internally, fibulae have equal spacing, there is no wider gap between centre fibulae. Distal raphe fissures terminate with small helictoglossa (Fig. 9E).

#### ﻿ Nitzschia alpinobacillum

Taxon classificationPlantaeCapsalideaCapsalidae

Lange-Bertalot, 1993

26CFC137-EC43-51B9-8854-EDF992A5656E

##### Notes.

**Figure 10.**
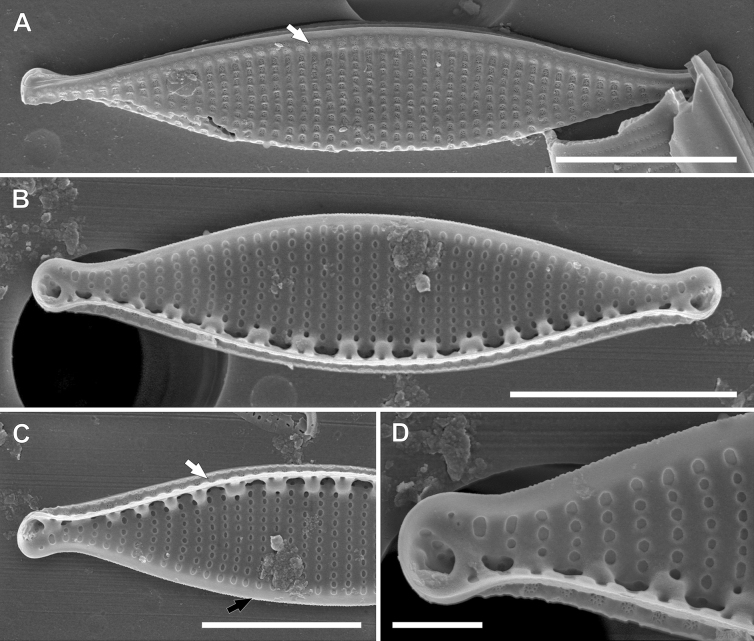
SEM photomicrographs of *Nitzschiaalpinobacillum***A** external view of the valve, two rows of areolae on the raphe canal, single and doubled areolae, covered by hymenes (white arrow), distal raphe fissure strongly deflected and extends on the proximal mantle **B** internal view of the valve, there is no wider extension between central fibulae **C, D** internal view of the apices **C** areolae on the proximal mantle are arranged the same as on the raphe canal: single and double areolae and covered by hymenes (white arrow), narrow distal mantle (black arrow) **D** distal raphe fissure terminates with helictoglossa. Scale bars: 5 µm (**A–C**); 1 µm (**D**).

The outline of the frustules is lanceolate, distended with capitate to acutely rounded apices (Fig. 3AX–BI). Valves are 15–22 µm length and 3.0–4.0 µm in width. The ratio of length and width is 4.21–6.41. Striae 23–25 in 10 µm visible in LM. Fibulae are visible 10–14 in 10 µm. Striae are uniseriate, arranged transapically. Areolae are covered by the hymenes. Along the edge of the valve, where the raised raphe canal is located, two longitudinal rows of areolae are present – a doublet nearer to the raphe opened to the interior of the raphe canal and a single areola at the junction with the valve face opened to the valve interior. (Fig. 10A, C–D). The distal mantle is narrow. Internally, fibulae have equal spacing; there is no wider gap between the centre fibulae (Fig. 10B). Distal raphe fissures terminate with small helictoglossa (Fig. 10D).

### ﻿nMDS analysis

**Figure 11.**
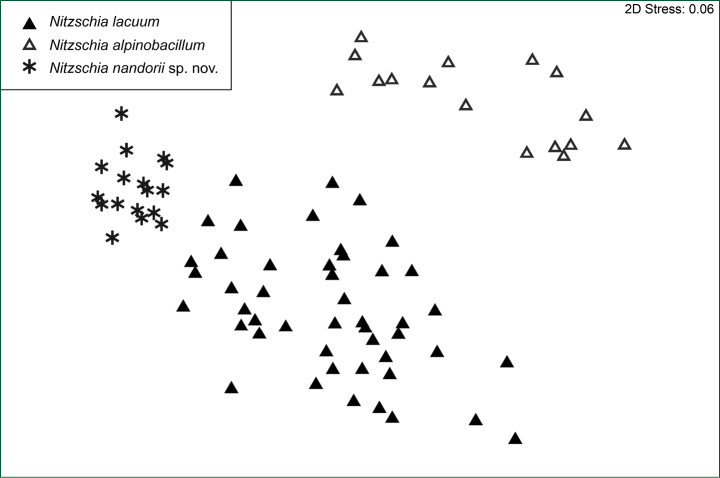
Two-dimensional nMDS plot. Analysis of valve morphology of three *Nitzschia* species based on the Euclidean distance.

nMDS analysis (stress value = 0.06) of 83 individuals revealed three different groups corresponding to three identified taxa (Fig. 11). The group labelled by asterisk corresponds to the newly described species *Nitzschianandorii* sp. nov. and is separate from the group labelled by full triangles. However, it is tightly distributed what may indicate low morphological variability. The group labelled by full triangles corresponds to the *N.lacuum* and is scattered which may indicate high morphological variability and the ambiguity of this taxa. The group labelled by empty triangles corresponds to *N.alpinobacillum*, and is scattered, which indicates high morphological variability.

## ﻿Discussion and conclusions

*Nitzschianandorii* sp. nov. is a typical representative of Grunow’s Lanceolatae section: lanceolate valve shape, strongly eccentric raphe system, and visible and easily distinguished fibulae (Cleve and Grunow 1880). Krammer and Lange-Bertalot (1997) indicated fibulae morphology as a trait which helps to identify *Nitzschia* species into two groups: one with a noticeable gap between the two centre fibulae and one without. However, this division is not reflected by recent molecular studies (Rimet et al. 2011; Mann et al. 2021), which suggest that the Lanceolatae section is more heterogeneous than it was presented in *Süβwasserflora von Mitteleuropa 2/2* (Krammer and Lange-Bertalot 1997). Mann et al. (2021) based on morphological data and a phylogenetic tree of the *rbcL* marker (ML) described a group of small *Nitzschia* that correspond to Lanceolate section, which possess two or more rows of poroids on the raphe canal that form a distinct clade. Our molecular analysis based on SSU, *rbcL*, *psbC* and concatenated dataset indicated that *N.nandorii* sp. nov. belongs to a clade with other small *Nitzschia* like *N.fonticola*, *N.acidoclinata*, *N.costei*, *N.soratensis*, and *N.perminuta*. Based on literature data all of these taxa were recognized with the same morphological trait i.e. two or more rows of poroids on the raphe canal. However, there are no central raphe fissures or the distinctive gap between central fibulae in *N.nandorii* sp. nov. and *N.perminuta* unlike *N.fonticola*, *N.acidoclinata*, *N.costei*, and *N.soratensis* (Morales and Vis 2007; Tudesque et al. 2008; Rovira et al. 2015; Mann et al. 2021; Bagmet et al. 2022). In the description of *N.lacuum*, Lange-Bertalot (1980) presented one specimen in fig. 95, plate 5 whose morphology corresponds to *N.nandorii* sp. nov. It has less protracted endings and the shape of the valve is more distended than other presented specimens of *N.lacuum*. Based on our observation, this specimen is *N.nandorii* sp. nov. Taken together, the morphometrical investigations, and morphological analyses of frustules and chloroplasts using SEM, LM and CLSM techniques, as well as phylogenetic studies based on a three-genes analysis allowed us to describe *Nitzschianandorii* sp. nov.

## Supplementary Material

XML Treatment for Nitzschia nandorii

XML Treatment for Nitzschia lacuum

XML Treatment for Nitzschia alpinobacillum

## ﻿Additional information

### Author ORCIDs

Rafał M. Olszyński https://orcid.org/0000-0003-0667-472X

Piotr K. Zakrzewski https://orcid.org/0000-0002-7205-8901

Frédéric Rimet https://orcid.org/0000-0002-5514-869X

Julia Sulkowska https://orcid.org/0009-0003-8456-1760

Łukasz Peszek https://orcid.org/0000-0002-9132-2210

Joanna Żelazna-Wieczorek https://orcid.org/0000-0003-1044-8151

### Data availability

All of the data that support the findings of this study are available in the main text or Supplementary Information.

## Supplementary materials

Supplementary material 1 Measurements of selected traits of *Nitzschia* spp from one environmental sample Data type xlsx https://binary.pensoft.net/file/1020068This dataset is made available under the Open Database License (http://opendatacommons.org/licenses/odbl/1.0/). The Open Database License (ODbL) is a license agreement intended to allow users to freely share, modify, and use this Dataset while maintaining this same freedom for others, provided that the original source and author(s) are credited.Author: Joanna Żelazna-Wieczorek

Supplementary material 2 List of 116 diatom taxa used in the phylogenetic analysis with GenBank accession numbers Data type docx https://binary.pensoft.net/file/1020069This dataset is made available under the Open Database License (http://opendatacommons.org/licenses/odbl/1.0/). The Open Database License (ODbL) is a license agreement intended to allow users to freely share, modify, and use this Dataset while maintaining this same freedom for others, provided that the original source and author(s) are credited.Author: Piotr K. Zakrzewski

Supplementary material 3 File with *psbC* alignment Data type fasta https://binary.pensoft.net/file/1020070This dataset is made available under the Open Database License (http://opendatacommons.org/licenses/odbl/1.0/). The Open Database License (ODbL) is a license agreement intended to allow users to freely share, modify, and use this Dataset while maintaining this same freedom for others, provided that the original source and author(s) are credited.Author: Piotr K. Zakrzewski

Supplementary material 4 File with *rbcL* alignment Data type fasta https://binary.pensoft.net/file/1020071This dataset is made available under the Open Database License (http://opendatacommons.org/licenses/odbl/1.0/). The Open Database License (ODbL) is a license agreement intended to allow users to freely share, modify, and use this Dataset while maintaining this same freedom for others, provided that the original source and author(s) are credited.Author: Piotr K. Zakrzewski

Supplementary material 5 File with SSU alignment Data type fasta https://binary.pensoft.net/file/1020072This dataset is made available under the Open Database License (http://opendatacommons.org/licenses/odbl/1.0/). The Open Database License (ODbL) is a license agreement intended to allow users to freely share, modify, and use this Dataset while maintaining this same freedom for others, provided that the original source and author(s) are credited.Author: Piotr K. Zakrzewski

Supplementary material 6 File with three-gene concatenated alignment Data type fasta https://binary.pensoft.net/file/1020073This dataset is made available under the Open Database License (http://opendatacommons.org/licenses/odbl/1.0/). The Open Database License (ODbL) is a license agreement intended to allow users to freely share, modify, and use this Dataset while maintaining this same freedom for others, provided that the original source and author(s) are credited.Author: Piotr K. Zakrzewski

Supplementary material 7 Partitioning data Data type txt https://binary.pensoft.net/file/1020074This dataset is made available under the Open Database License (http://opendatacommons.org/licenses/odbl/1.0/). The Open Database License (ODbL) is a license agreement intended to allow users to freely share, modify, and use this Dataset while maintaining this same freedom for others, provided that the original source and author(s) are credited.Author: Piotr K. Zakrzewski

Supplementary material 8 Confocal Laser Scanning Microscopy projection of rotating chloroplast of *Nitzschianandorii* sp. nov. Data type gif https://binary.pensoft.net/file/1020075This dataset is made available under the Open Database License (http://opendatacommons.org/licenses/odbl/1.0/). The Open Database License (ODbL) is a license agreement intended to allow users to freely share, modify, and use this Dataset while maintaining this same freedom for others, provided that the original source and author(s) are credited.Author: Rafał M. Olszyński

Supplementary material 9 SEM photographs of *N.nandorii* form culture Data type jpg Explanation note **A** valve view, the white arrow indicates a lack of central raphe fissures **B** valve view **C** apice view, the white arrow indicates a row of areolae on the proximal mantle. https://binary.pensoft.net/file/1020076This dataset is made available under the Open Database License (http://opendatacommons.org/licenses/odbl/1.0/). The Open Database License (ODbL) is a license agreement intended to allow users to freely share, modify, and use this Dataset while maintaining this same freedom for others, provided that the original source and author(s) are credited.Author: Łukasz Peszek

Supplementary material 10 Maximum likelihood phylogenetic tree of *Nitzschia* spp. based on the *psbC* molecular marker Data type jpg https://binary.pensoft.net/file/1020077This dataset is made available under the Open Database License (http://opendatacommons.org/licenses/odbl/1.0/). The Open Database License (ODbL) is a license agreement intended to allow users to freely share, modify, and use this Dataset while maintaining this same freedom for others, provided that the original source and author(s) are credited.Author: Rafał M. Olszyński

Supplementary material 11 Bayesian Inference phylogenetic tree of *Nitzschia* spp. based on the *psbC* molecular marker Data type jpg https://binary.pensoft.net/file/1020078This dataset is made available under the Open Database License (http://opendatacommons.org/licenses/odbl/1.0/). The Open Database License (ODbL) is a license agreement intended to allow users to freely share, modify, and use this Dataset while maintaining this same freedom for others, provided that the original source and author(s) are credited.Author: Rafał M. Olszyński

Supplementary material 12 Maximum likelihood phylogenetic tree of *Nitzschia* spp. based on the SSU rDNA molecular marker Data type jpg https://binary.pensoft.net/file/1020079This dataset is made available under the Open Database License (http://opendatacommons.org/licenses/odbl/1.0/). The Open Database License (ODbL) is a license agreement intended to allow users to freely share, modify, and use this Dataset while maintaining this same freedom for others, provided that the original source and author(s) are credited.Author: Rafał M. Olszyński

Supplementary material 13 Bayesian Inference phylogenetic tree of *Nitzschia* spp. based on the SSU rDNA molecular marker Data type jpg https://binary.pensoft.net/file/1020080This dataset is made available under the Open Database License (http://opendatacommons.org/licenses/odbl/1.0/). The Open Database License (ODbL) is a license agreement intended to allow users to freely share, modify, and use this Dataset while maintaining this same freedom for others, provided that the original source and author(s) are credited.Author: Rafał M. Olszyński
