# Supplementary material for: ﻿Morphology and phylogeny of Nitzschianandorii sp. nov. (Bacillariophyceae), a new small-celled lanceolate species from a post-mining reservoir
Source: PhytoKeys. 2024 Apr 4;241:1–26. doi: 10.3897/phytokeys.241.117406 (PMC11009488; doi:10.3897/phytokeys.241.117406)
Supplement: Supplementary material 2 — List of 116 diatom taxa used in the phylogenetic analysis with GenBank accession numbers [file phytokeys-241-001_article-117406__-s002.docx]

**Table S1.** List of 116 diatom taxa used in the phylogenetic analysis with GenBank accession numbers. Bolded text used for new taxa/new submitted sequences.

| **Taxon** | **Strain** | **GenBank accession numbers** | | |
| --- | --- | --- | --- | --- |
|  |  | **SSU** | ***rbcL*** | ***psbC*** |
| *Eunotia bilunaris* | UTEX FD412 | HQ912599 | HQ912463 | HQ912292 |
| *Nitzschia acicularis* | R20 | MN750494 | KX889095 | MN734050 |
| *Nitzschia acidoclinata* | TCC:560 | MN696711 | MN696765 | - |
| *Nitzschia adhaerens* | BIOTAII-18 | MH734165 | MH687900 | MH687889 |
| *Nitzschia aequorea* | Dillu38 | KY320391 | KY320330 | - |
| *Nitzschia* aff. *sublinearis* | BC0850 | - | MN718797 | - |
| *Nitzschia alicae* | BC0330 | - | MN718762 | - |
| *Nitzschia amphibia* | TCC:498 | MN696707 | MN696761 | - |
| *Nitzschia amphibia* | RT5 | - | HF675118 | - |
| *Nitzschia amphibia* | BC0701 | - | MN718781 | - |
| *Nitzschia asteropeae* | GU52V2 oblongB7 | - | MW324605 | MW324656 |
| *Nitzschia bergii* | TA139 | KY320379 | KY320318 | - |
| *Nitzschia capitellata* | BC0713 | - | MN718783 | - |
| *Nitzschia capitellata* | Scot1 | MN750453 | FN557030 | MN734010 |
| *Nitzschia captiva* | IRTACC152 | - | LC482715 | LC746294 |
| *Nitzschia* cf. *aequorea* | LRT-2013 DM1004CAT | MN750480 | HF675062 | MN734034 |
| *Nitzschia* cf. *ardua* | LRT-2013 L44 | MN750476 | HF675061 | MN734030 |
| *Nitzschia* cf. *bulnheimiana* | LRT-2013 AG | MN750449 | HF675063 | - |
| *Nitzschia* cf. *dissipata var. media* | BC0470 | MN750422 | MN718767 | - |
| *Nitzschia* cf. *dubiiformis* | SZCZCH970 | - | KT943666 | KT943701 |
| *Nitzschia* cf. *fonticola* | LRT-2013 cf. fonticola 1 | - | HF675064 | - |
| *Nitzschia* cf. *fonticola* | BC0053 | - | MN718749 | - |
| *Nitzschia* cf. *frigida* | AKIce Nitz | - | MH064110 | MH064017 |
| *Nitzschia* cf. *frustulum* | TCC521 | KC736634 | KC736604 | - |
| *Nitzschia* cf. *gracilis* | Nit51 | MN750487 | MN734082 | MN734043 |
| *Nitzschia* cf. *hantzschiana* | NIT337TM | - | MN734074 | - |
| *Nitzschia* cf. *longissima* | Cylin clos | - | MN734068 | - |
| *Nitzschia* cf. *microcephala* | LRT-2013 L56 | MN750477 | HF675103 | - |
| *Nitzschia* cf. *palea* | BC0799 | - | MN718792 | - |
| *Nitzschia* cf. *paleacea* | BC0675 | - | MN718779 | - |
| *Nitzschia* cf. *paleacea* | BC0806 | - | MN718793 | - |
| *Nitzschia* cf. *paleacea* | TA406 | KY320380 | KY320319 | - |
| *Nitzschia* cf. *perminuta* | BC0730 | - | MN718784 | - |
| *Nitzschia* cf. *perminuta* | BC0838 | - | MN718795 | - |
| *Nitzschia* cf. *pusilla* | TCC:665 | MN696723 | MN696772 | - |
| *Nitzschia* cf. *pusilla* | CCMP558 | MN750456 | HF675129 | MN734013 |
| *Nitzschia* cf. *pusilla* | NIT1003CAT | MN750479 | MN734077 | MN734033 |
| *Nitzschia* cf. *pusilla* | Nit44 | MN750485 | HF675119 | MN734041 |
| *Nitzschia* cf. *recta* | BC0769 | - | MN718786 | - |
| *Nitzschia* cf. *recta* | BC0795 | MN750424 | MN718791 | - |
| *Nitzschia* cf. *romana* | BC0650 | - | MN718776 | - |
| *Nitzschia* cf. *sigma* | BC0308 | - | MN718759 | - |
| *Nitzschia* cf. *sigma* | NIT1013ABR | MN750484 | MN734080 | MN734038 |
| *Nitzschia* cf. *sigmoidea* | KEL-2015 JAR89_G2Run12 | - | KM999113 | - |
| *Nitzschia* cf. *soratensis* | BC0501 | - | MN718769 | - |
| *Nitzschia costei* | BC0469 | - | MN718766 | - |
| *Nitzschia dalmatica* | PMFBION3 | - | MH687910 | MH687899 |
| *Nitzschia dalmatica* | PMFBIONA1 | - | MH687909 | MH687898 |
| *Nitzschia dissipata* | TCC:707 | MN696724 | MN696773 | - |
| *Nitzschia dissipata* | SZCZCH845 | - | KT943665 | - |
| *Nitzschia dissipata* | TA44 | KY320393 | KY320332 | - |
| *Nitzschia dissipata* var. *media* | BC0649 | - | MN718775 | - |
| *Nitzschia draveillensis* | TCC700 | KC736635 | KC736605 | - |
| *Nitzschia draveillensis* | BC0325 | - | MN718761 | - |
| *Nitzschia draveillensis* | Nit50 | MN750486 | MN734081 | MN734042 |
| *Nitzschia dubia* | TA37 | KY320381 | KY320320 | - |
| *Nitzschia dubiiformis* | s0311 | AB430616 | AB430696 | - |
| *Nitzschia filiformis* | UTEX FD267 | HQ912589 | HQ912453 | HQ912282 |
| *Nitzschia fonticola* | A-RT24 | - | HF675066 | - |
| *Nitzschia fonticola* | C-RT26 | MN750452 | HF675068 | - |
| *Nitzschia frigida* | AK | ON783211 | ON791849 | - |
| *Nitzschia frustulum* | TCC:550 | MN696710 | MN696764 | - |
| *Nitzschia frustulum* | CCMP558 | - | EF423498 | EF520303 |
| *Nitzschia frustulum* | Nit25 | - | HF675070 | MN734040 |
| *Nitzschia gracilis* | TCC:576 | MN696715 | MN696768 | - |
| *Nitzschia heufleriana* | BC0307 | - | MN718758 | - |
| *Nitzschia inconspicua* | SZCZE461 | - | MK454986 | - |
| *Nitzschia inconspicua* | G1_1 | - | HF675071 | - |
| *Nitzschia inconspicua* | G7_1 | MN750470 | MN734076 | - |
| *Nitzschia inordinata* | BIOTAII-44 | MH734171 | MH687906 | MH687895 |
| *Nitzschia lembiformis* | R2 | MN750493 | HE802701 | MN734049 |
| *Nitzschia liebethruthii* | TA353 | KY320378 | KY320317 | - |
| *Nitzschia ligowskii* | TA426 | KY320392 | KY320331 | - |
| *Nitzschia linearis* | BC0083 | - | MN718751 | - |
| *Nitzschia linearis* | Nit53 | MN750488 | MN734083 | MN734044 |
| *Nitzschia longissima* | KSA2015-9 Nitz.longi-ED | MH063481 | MH064112 | MH064019 |
| *Nitzschia lorenziana* | TCC516 | KC736637 | KC736608 | - |
| *Nitzschia microcephala* | R10 | MN750491 | MN734086 | MN734047 |
| ***Nitzschia nandorii* sp. nov.** | **D.LDZ8** | **PP082029** | **PP073739** | **PP073738** |
| ***Nitzschia nandorii* sp. nov.** | **D.LDZ12** | **PP082030** | **PP073741** | **PP073740** |
| *Nitzschia nanodissipata* | SZCZCH974 | - | KT943675 | - |
| *Nitzschia palea* | TCC:945 | - | MN696782 | - |
| *Nitzschia palea* | BB2b | - | KJ542517 | - |
| *Nitzschia palea* | DM1011 | - | KJ542490 | - |
| *Nitzschia palea* | TCC 139-3 | - | KJ542520 | - |
| *Nitzschia palea* | TCC 651 | - | KJ542478 | - |
| *Nitzschia palea* | NIT1011KEL [DM1011] | - | MN734078 | - |
| *Nitzschia palea* | R8 | - | MN734087 | - |
| *Nitzschia palea* | TCC909 | KY799131 | KY799144 | - |
| *Nitzschia paleacea* | BC0483 | - | KX889092 | - |
| *Nitzschia paleaeformis* | TA394 | KY320383 | KY320322 | - |
| *Nitzschia pellucida* | EW229 | KY320389 | KY320328 | - |
| *Nitzschia perminuta* | TCC:885 | MN696726 | MN696777 | - |
| *Nitzschia pusilla* | BC0333 | - | MN718763 | - |
| *Nitzschia pusilla* | L1 | MN750473 | HF675108 | MN734027 |
| *Nitzschia pusilla* | TA-45 | KY320384 | KY320323 | - |
| *Nitzschia pusilla* | TCC898 | KY863480 | KY863494 | - |
| *Nitzschia rectilonga* | SZCZE431 | MN943999 | MN920679 | MN920688 |
| *Nitzschia schefterae* | 19X15-1B thinraphidB11 | - | MW324606 | MW324657 |
| *Nitzschia schefterae* | GU52X-1 NitzC28 | - | MW324607 | MW324658 |
| *Nitzschia sigma strain* | TA341 | KY320395 | KY320337 | - |
| *Nitzschia sigma* | TA377 | KY320385 | KY320324 | - |
| *Nitzschia sigmaformis* | TA311 | KY320386 | KY320325 | - |
| *Nitzschia sigmoidea* | BC0787 | MN750423 | MN718790 | - |
| *Nitzschia sigmoidea* | RBGE | - | FN557033 | - |
| *Nitzschia soratensis* | DM1008MK | MN750481 | HF675111 | - |
| *Nitzschia supralitorea* | TCC:944 | - | MN696781 | - |
| *Nitzschia supralitorea* | TCC:946 | - | MN696783 | - |
| *Nitzschia supralitorea* | NIT145D | - | MN734070 | - |
| *Nitzschia taygeteae* | GU52X-1 NitzED21 | - | MW324615 | MW324665 |
| *Nitzschia tubicola* | TCC:575 | MN696714 | MN696767 | - |
| *Nitzschia umbonata* | NIT327TM | - | MN734071 | - |
| *Nitzschia valdestriata* | SZCZCH969 | KT943640 | KT943664 | KT943699 |
| *Nitzschia varelae* | Nit952CAT | MN750490 | KX889093 | MN734046 |
| *Nitzschia volvendirostrata* | CCMP2144 | - | MW324612 | MW324662 |
| *Nitzschia volvendirostrata* | SZCZP36 | - | KU179114 | KU179141 |
