# Supplementary figures and images for: ﻿Morphology and phylogeny of Nitzschianandorii sp. nov. (Bacillariophyceae), a new small-celled lanceolate species from a post-mining reservoir
Source: PhytoKeys. 2024 Apr 4;241:1–26. doi: 10.3897/phytokeys.241.117406 (PMC11009488; doi:10.3897/phytokeys.241.117406)

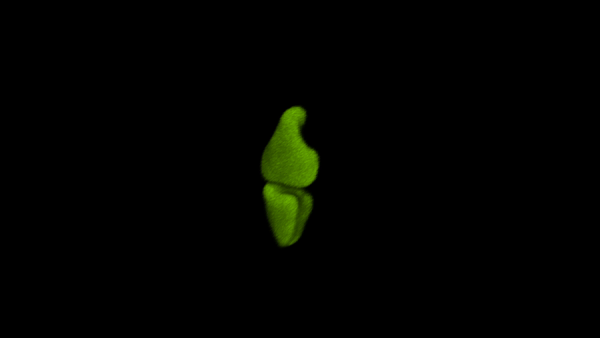

Supplement: Supplementary material 8 — Confocal Laser Scanning Microscopy projection of rotating chloroplast of Nitzschianandorii sp. nov. [file phytokeys-241-001_article-117406__-s008.gif]

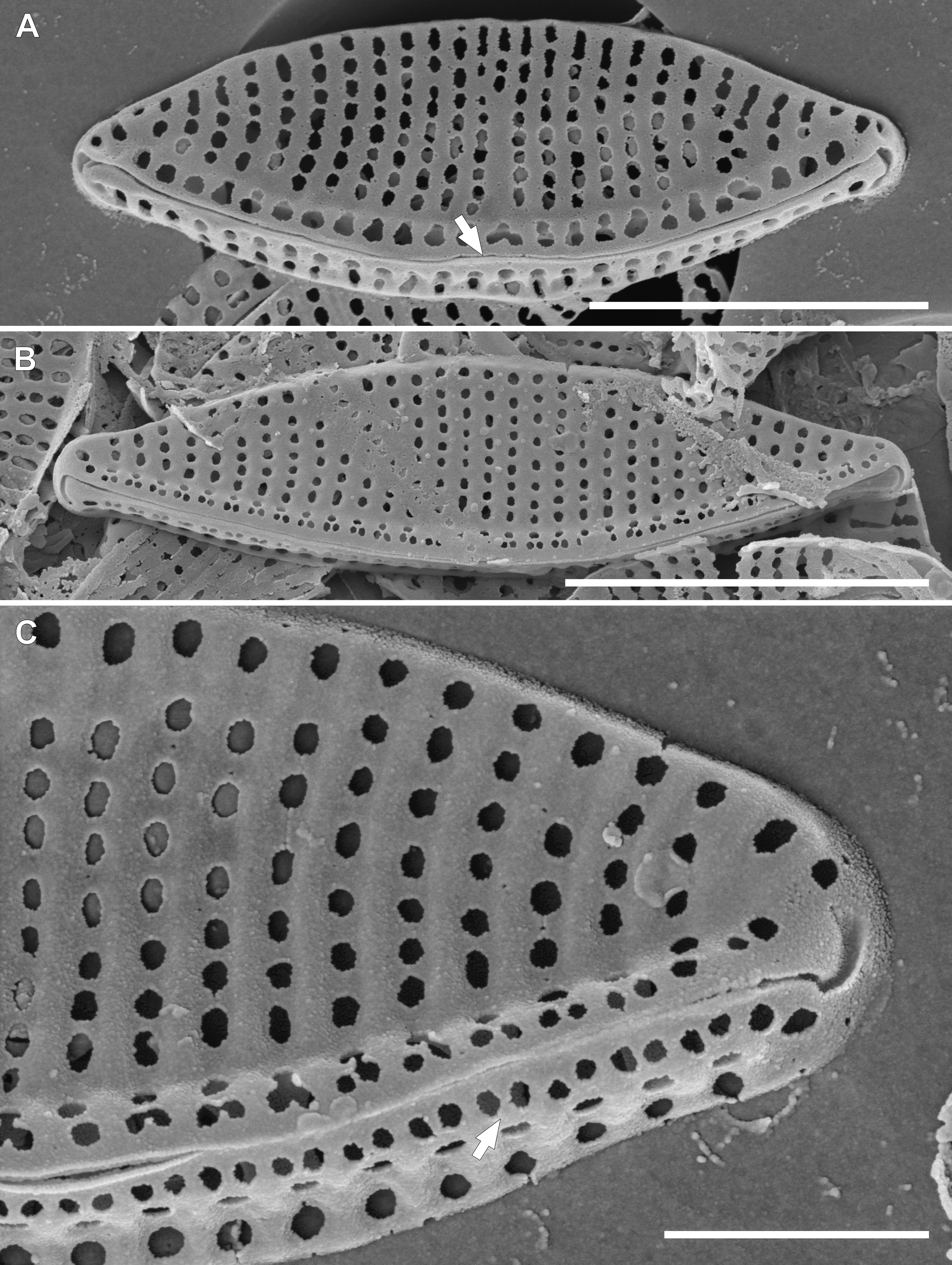

Supplement: Supplementary material 9 — SEM photographs of N.nandorii form culture [file phytokeys-241-001_article-117406__-s009.jpg]

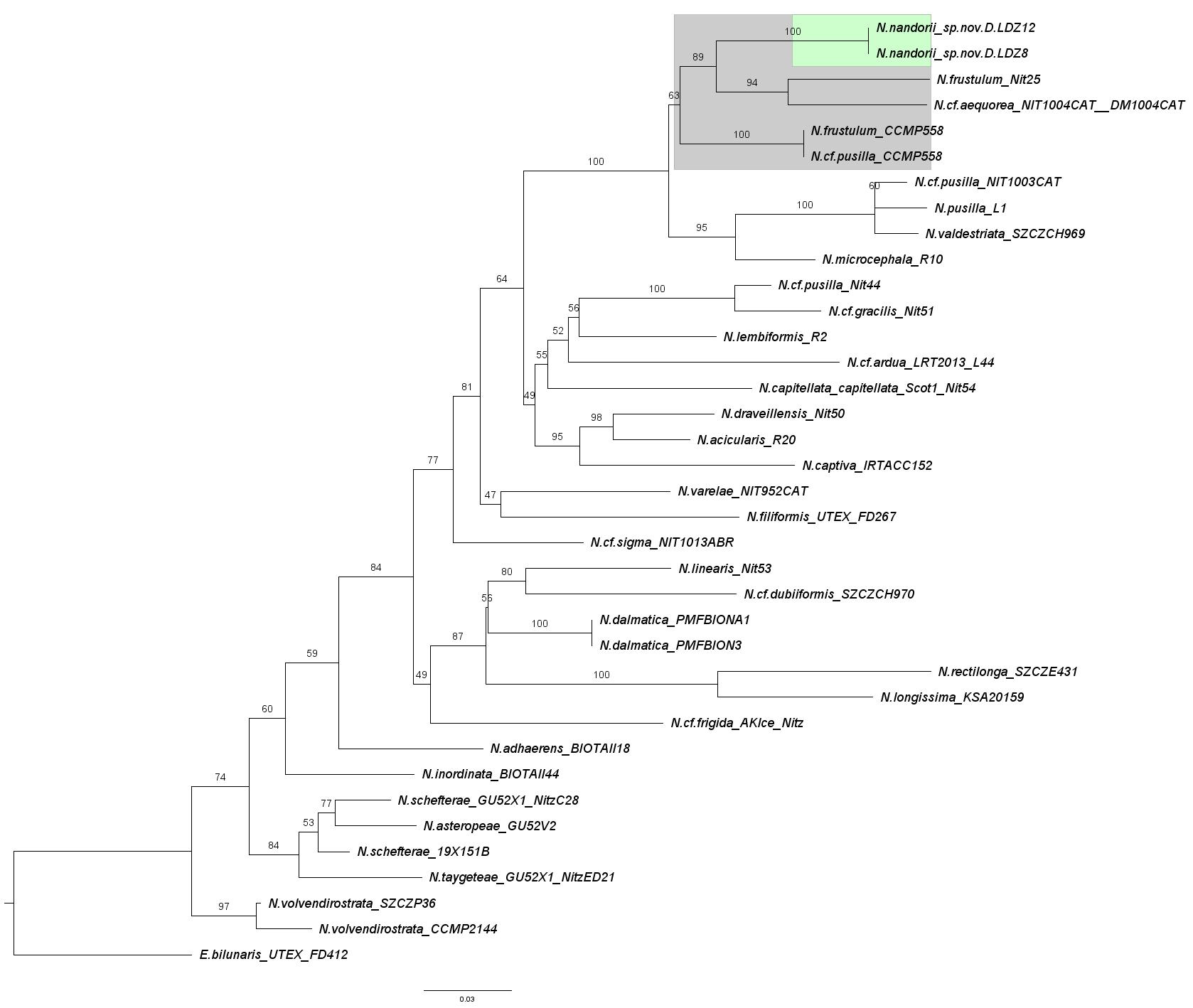

Supplement: Supplementary material 10 — Maximum likelihood phylogenetic tree of Nitzschia spp. based on the psbC molecular marker [file phytokeys-241-001_article-117406__-s010.jpg]

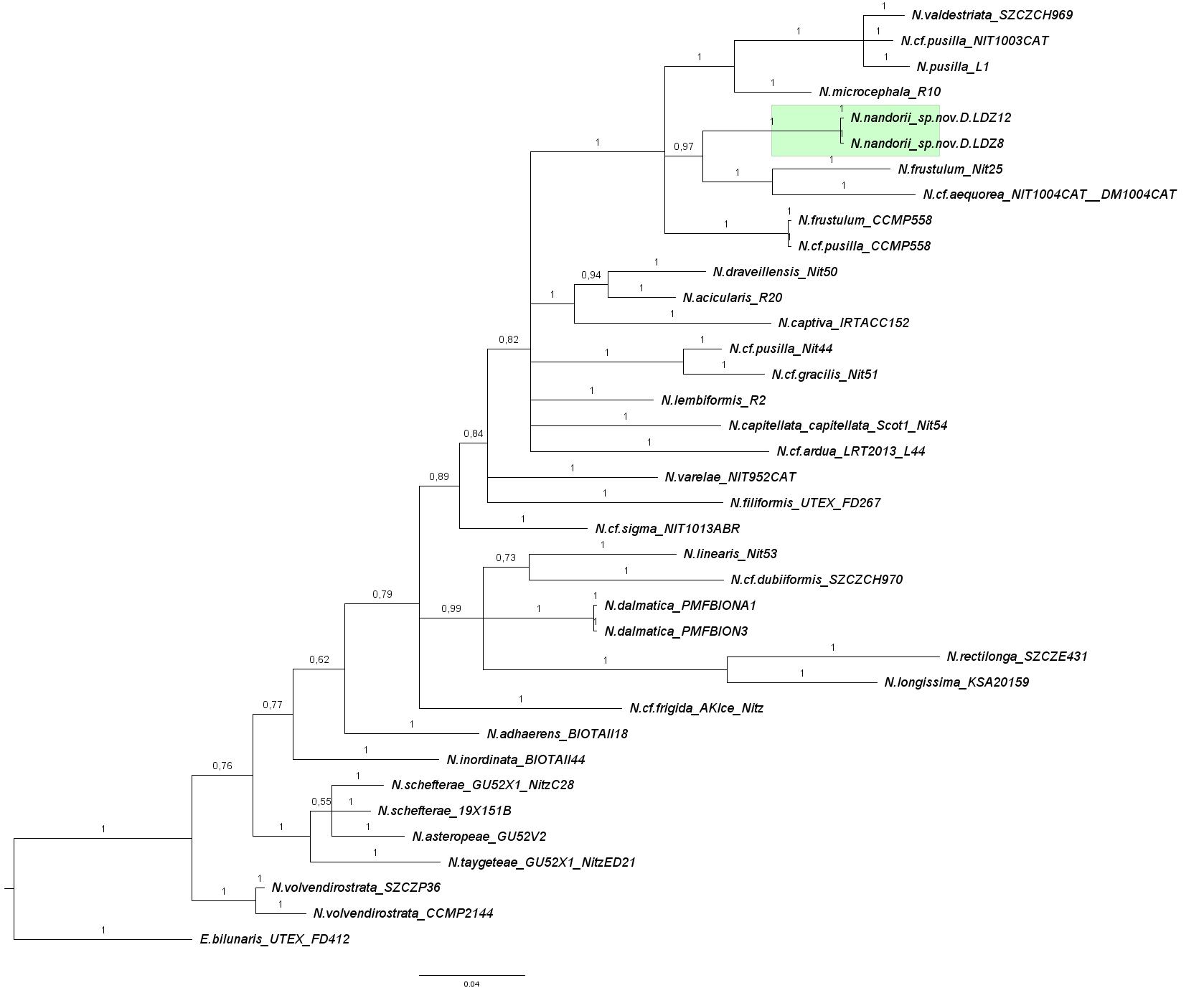

Supplement: Supplementary material 11 — Bayesian Inference phylogenetic tree of Nitzschia spp. based on the psbC molecular marker [file phytokeys-241-001_article-117406__-s011.jpg]

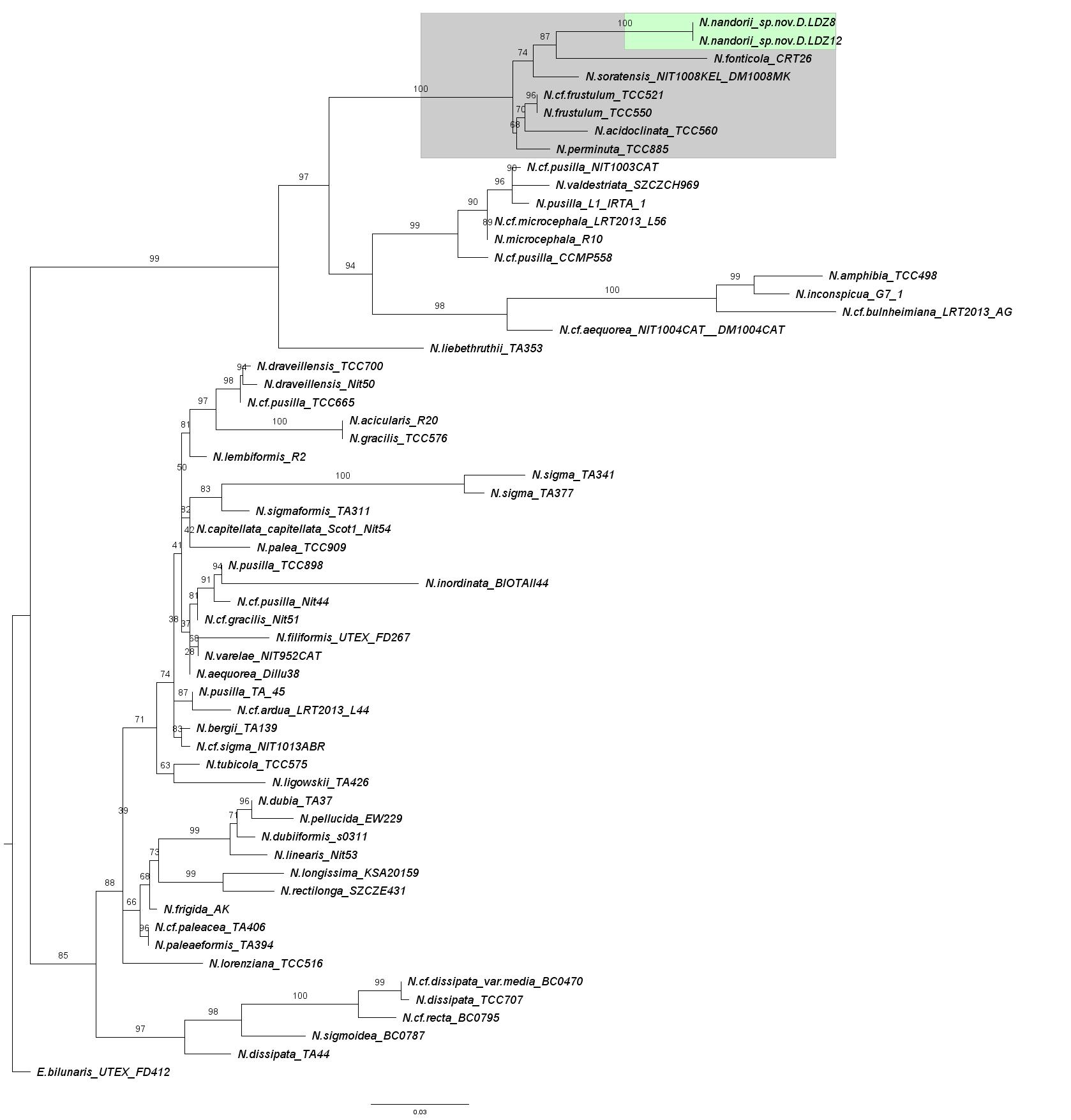

Supplement: Supplementary material 12 — Maximum likelihood phylogenetic tree of Nitzschia spp. based on the SSU rDNA molecular marker [file phytokeys-241-001_article-117406__-s012.jpg]

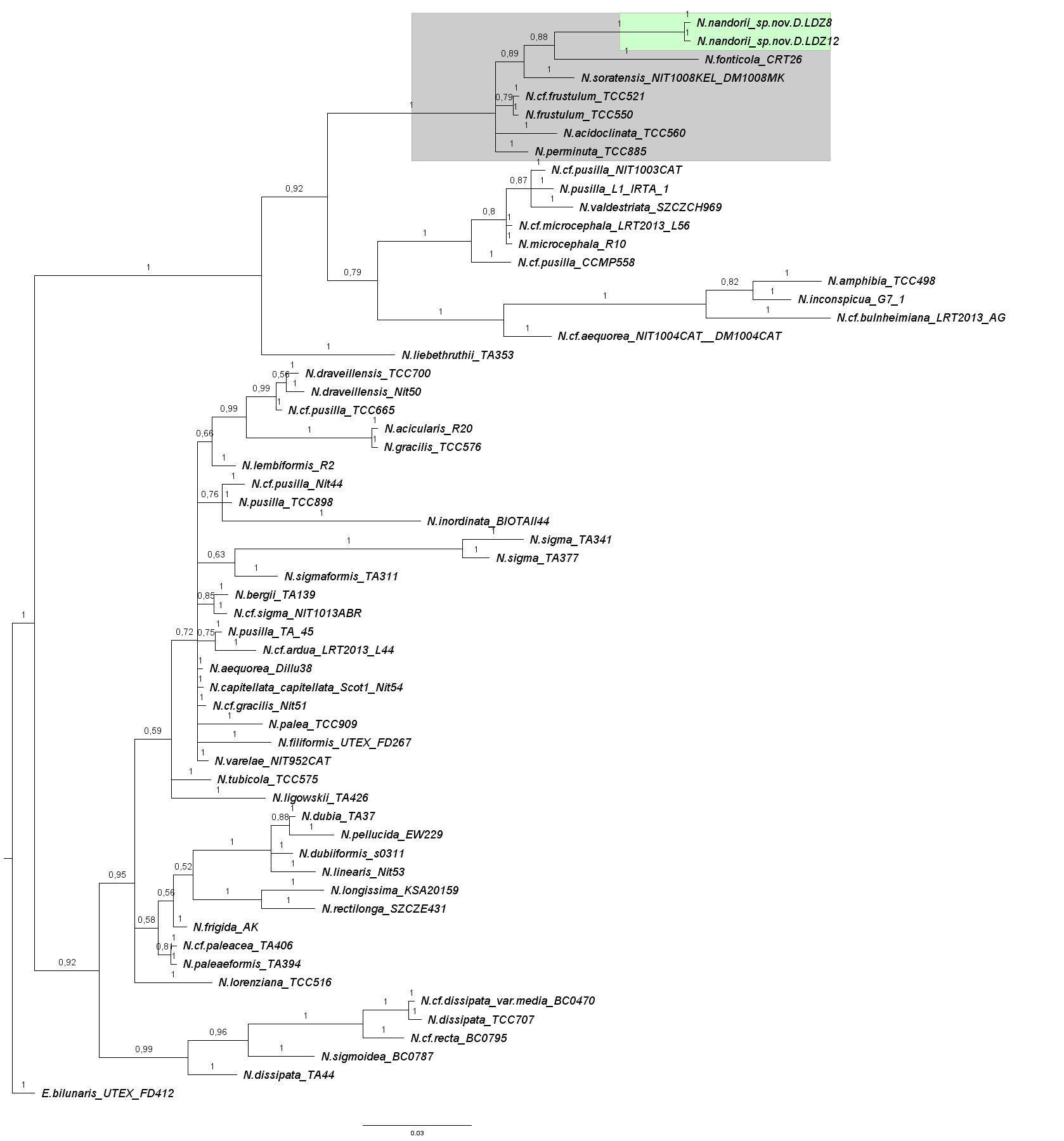

Supplement: Supplementary material 13 — Bayesian Inference phylogenetic tree of Nitzschia spp. based on the SSU rDNA molecular marker [file phytokeys-241-001_article-117406__-s013.jpg]
